# Enhancing Cytosolic Internalization of [^177^Lu]Lu–iPSMA in Prostate Cancer Cells: The Effect of Conjugating a GRP78 Inhibitor to the Radiotherapeutic Molecule

**DOI:** 10.3390/ijms262411783

**Published:** 2025-12-05

**Authors:** Erika Azorín-Vega, Daniel García-Arce, Myrna Luna-Gutiérrez, Blanca Ocampo-García, Diana Trujillo-Benítez, Abraham Vidal-Limon, Griselda Rodríguez-Martínez, María Luisa Durán-Pastén, Laura Meléndez-Alafort, Guillermina Ferro-Flores

**Affiliations:** 1 Department of Radioactive Materials, Instituto Nacional de Investigaciones Nucleares (ININ), Ocoyoacac 52750, Mexico; 2 Network for Advanced Molecular Studies, Instituto de Ecología A.C., Xalapa-Enríquez 91070, Mexico; 3 Respiratory Pathogens Research Laboratory, Hospital Infantil de México Federico Gómez, Mexico City 06720, Mexico; 4 Channelopathies National Laboratory, Cellular Physiology Institute, UNAM, Mexico City 04510, Mexico; 5 Immunology and Molecular Oncology Diagnostics Unit, Veneto Institute of Oncology IOV–IRCCS, 35128 Padua, Italy

**Keywords:** lutetium-177, GRP78 inhibitor, PSMA inhibitor, [^177^Lu]Lu–iPSMA

## Abstract

Castration-resistant prostate cancer presents radiotherapeutic challenges, especially in optimizing the cytosolic internalization of therapeutic radiopharmaceuticals. This research aimed to design and evaluate in vitro, a new dimeric radiopharmaceutical, [^177^Lu]Lu–iPSMA–iGRP78, which combines PSMA and GRP78 inhibitors in a heterodimeric radioligand to improve the radionuclide internalization and cytotoxicity efficacy. Molecular docking showed that the dimer iPSMA–iGRP78 presents a higher affinity for GRP78 (CNN-docking score: −14.0 kcal·mol^−1^, pKi: 10) and for PSMA (CNN-docking score: −17.0 kcal·mol^−1^, pKi: 11.5) compared to the monomers iGRP78 (CNN-docking score: −11.0 kcal·mol^−1^, pKi: 9.4) and iPSMA (CNN-docking score: −13.9 kcal·mol^−1^, pKi: 10.2). The saturation binding assay using LNCaP cells (PSMA+, CS-GRP78+) showed an affinity (K_d_) of 1.883 nM for [^177^Lu]Lu–iPSMA–iGRP78 and 2.245 nM for [^177^Lu]Lu–iPSMA. The dimeric radiopharmaceutical achieved 10.44 ± 2.43% cytosolic internalization and 4.81 ± 0.94% nuclear internalization, while the [^177^Lu]Lu–iPSMA monomer showed 6.45 ± 0.60% cytosolic internalization and no uptake in the cell nucleus. In PC3 cells (PSMA–, CS-GRP78–), [^177^Lu]Lu–iPSMA–iGRP78 uptake was negligible, demonstrating specificity. Treatment with the dimeric radiopharmaceutical reduced cell viability (69.93 ± 4.85% of dead cells) significantly more than [^177^Lu]Lu–iPSMA (38.63 ± 6.13% of dead cells). In conclusion, conjugation of a GRP78 inhibitor to [^177^Lu]Lu–iPSMA improves the radionuclide internalization and cytotoxicity in prostate cancer cells, suggesting that the bispecific radiopharmaceutical is a promising strategy in prostate cancer treatment.

## 1. Introduction

Targeted radiotherapies using lutetium-177-labeled prostate-specific membrane antigen inhibitors ([^177^Lu]Lu–iPSMA) have shown significant advancements in the treatment of metastatic castration-resistant prostate cancer [1,2,3,4].

In general, the PSMA inhibitor small molecules are internalized into prostate cancer cells via clathrin-mediated endocytosis after binding to PSMA on the cell membrane. During this process, the inhibitors accumulate in endocytic vesicles and are subsequently distributed homogeneously in the cytoplasm [5]. However, in the radiopharmaceutical form, iPSMA must be conjugated with the macrocycle DOTA (1,4,7,10-tetraazacyclododecane-N,N′,N″,N‴-tetraacetic acid) as a radionuclide chelator for labeling with ^177^Lu, even though DOTA has been reported to cause low internalization of iPSMA radioligands [6].

A high level of cytosolic internalization of [^177^Lu]Lu–iPSMA in prostate cancer cells is desirable to concentrate the radionuclide within the cytoplasm and increase the radiation-mediated damage to cancer cells. Attempts to improve the cytoplasmic internalization of PSMA ligands for ^177^Lu therapy involve a combination of epigenetic stimulation, dual-targeting ligands, enhanced receptor density and recycling, and advanced nanoparticle delivery systems [7,8,9,10,11,12,13]. Nevertheless, optimizing the internalization of PSMA radiopharmaceuticals remains a challenge to ensure maximum therapeutic benefit.

Stimuli from outside the cell or from within can cause problems with how proteins fold in the cell’s endoplasmic reticulum (ER). These problems can be caused by calcium disturbances inside the cell, low oxygen levels, not enough nutrients, low pH, reactive oxygen species, and signals that cause cell growth and inflammation. This results in the buildup of new or misfolded proteins in the ER, leading to a loss of proteostasis, also known as ER stress. Glucose-regulated protein 78 (GRP78), a member of the heat shock protein family (Hsp70), is a master chaperone molecule in the ER that participates in protein folding and assembly. It does so by binding to Ca^2+^ and maintaining ER homeostasis. When the ER is stressed, GRP78 is exported to the cytoplasm and cell membrane. There, it becomes exposed and is known as cell surface GRP78 (CS-GRP78) [14].

Cancer cells are exposed to nutritional deficiencies, hypoxia, acidosis, and increased protein synthesis, all of which cause sustained stress. To alleviate ER stress, malignant cells increase the expression of ER chaperones, including GRP78, which helps cancer cells escape oxidative stress-induced damage and cell death, thereby contributing to tumor development [15]. CS-GRP78 has been shown to form complexes with other proteins that perform a variety of functions in regulation of innate and adaptive immunity, apoptosis, cell viability, and proliferation. There is growing evidence that CS-GRP78 overexpression induces aggressive tumor behavior in various types of cancer, such as ovary, breast, pancreas, colon, and prostate [16]. In fact, CS-GRP78 is considered a functional molecular target on the cell surface in the development of therapies for androgen-independent metastatic prostate cancer [17]. Targeting CS-GRP78 in tumor cells effectively reduces radio- and chemo-resistance [18,19]. Some of these therapies use GRP78-inhibiting peptide sequences conjugated to drugs that bind to the GRP78 ATPase domain, allowing for subsequent internalization into the cell cytoplasm [20,21,22,23]. In particular, the amino acid sequence WIFPWIQL (iGRP78) has been predicted to bind GRP78 specifically and exhibits excellent affinity as a GRP78 inhibitor peptide [20].

This study aimed to estimate the molecular interactions and affinity score of a new heterodimeric radiopharmaceutical, [^177^Lu]Lu–iPSMA–iGRP78 ([^177^Lu]Lu–DOTA–HYNIC–iPSMA–iGRP78), in silico, and to evaluate its potential to increase cytosolic internalization in prostate cancer cells in vitro compared to the monomeric radiopharmaceutical [^177^Lu]Lu–iPSMA ([^177^Lu]Lu–DOTA–HYNIC–iPSMA).

## 2. Results and Discussion

**Figure A1:**
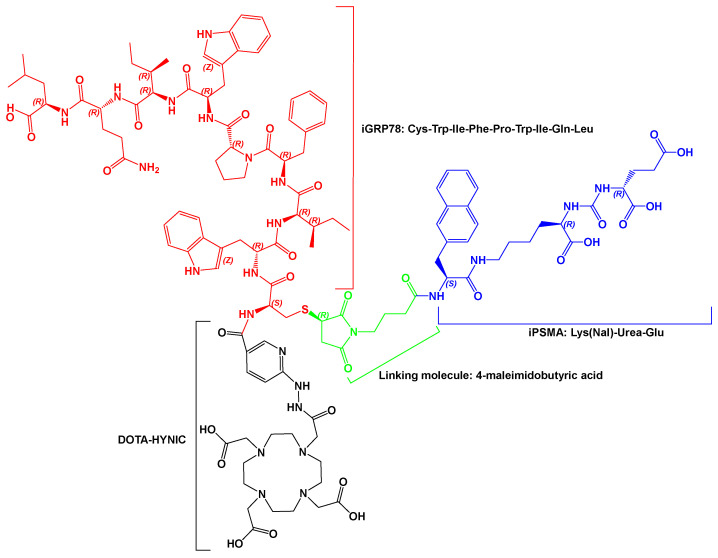
Schematic chemical structure of the novel DOTA-HYNIC–iPSMA–iGRP78 dimeric peptide ligand, with DOTA–HYNIC at the center of the iPSMA and iGRP78 sequences connected via a 4-maleimidobutyric acid, which forms the branching point (4-(2,5-dioxopyrrolidin-1-yl)butanal).

**Figure A2:**
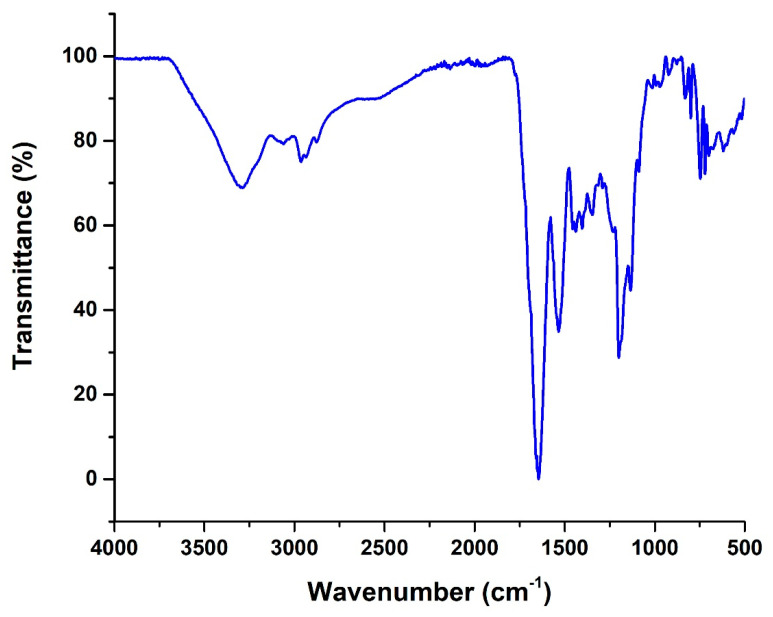
FT-IR analysis (500–4000 cm^−1^, 20 scans at 0.4 cm^−1^) of DOTA-HYNIC–iPSMA–iGRP78 was performed using a Perkin Elmer System 2000 spectrometer equipped with an Attenuated Total Reflection Platform (PerkinElmer, Waltham, MA, USA). The peak attributed to the -C-O- stretching from DOTA carboxylic acids (O=*CO*-H) is observed at 1205 cm^−1^, while the C=O bonds of the peptide were located at 1646 and 1437 cm^−1^. The band at 720 cm^−1^ was attributed to the C-S group associated with the maleimidopropyl group. Peaks located at 2963 cm^−1^, and 2937 cm^−1^ were associated with –CH_2_– bonds. The -C=O motif contribution from the amides in the structure was observed at 1646 and 1661 cm^−1^. The stretching vibrational band at 2937 correlated with νC-H bonds. The C-N tertiary amines were observed at 1134 cm^−1^.

**Figure A3:**
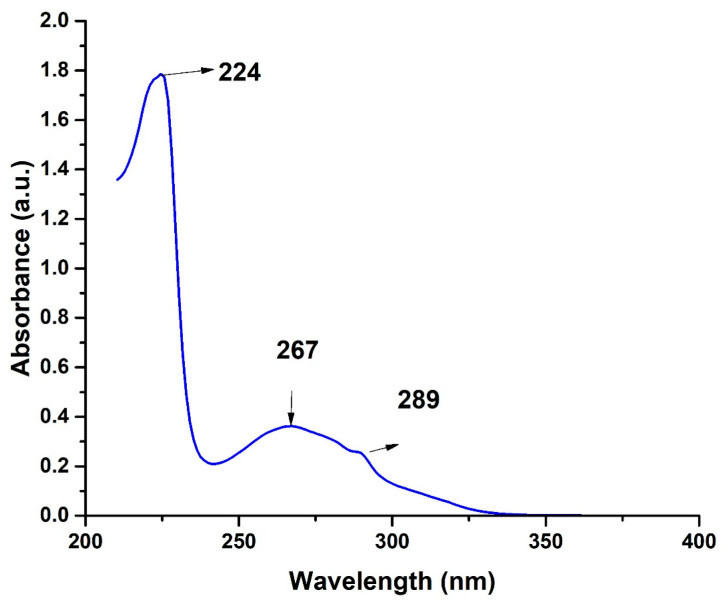
The ultraviolet absorption spectra of DOTA-HYNIC–iPSMA–iGRP78 were recorded using a PerkinElmer Lambda Bio spectrophotometer (200–400 nm) (PerkinElmer, Waltham, MA, USA). The π ⟶ π*transitions were observed at 232 nm. The n ⟶ σ* transition from the amine groups was observed in a band centered at 224 nm, associated with the nonbonding electron pairs. The 267 nm wavelength indicates the presence of the C-S bond. In addition, the n ⟶ σ* transition of -C=O was observed at 289 nm.

**Figure A4:**
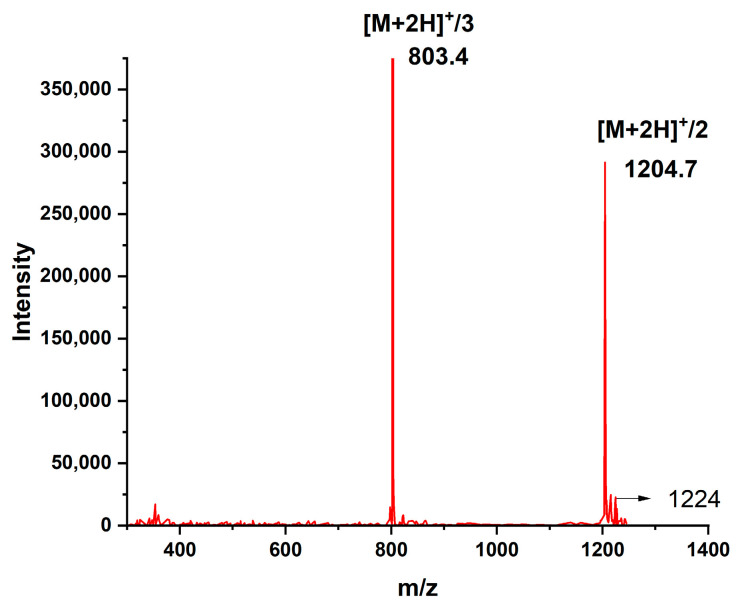
The mass spectra of DOTA-HYNIC–iPSMA–iGRP78 showed the corresponding signals at *m*/*z* 803 (cal. 804) [M + 2H]^+^/3 and *m*/*z* 1204 (cal. 1205) [M + 2H]^+^/2. Analysis was performed on an ADQUITY UPLC H-Class equipped with a QDa mass detector (Waters Corporation, Milford, CT, USA).

**Figure A5:**
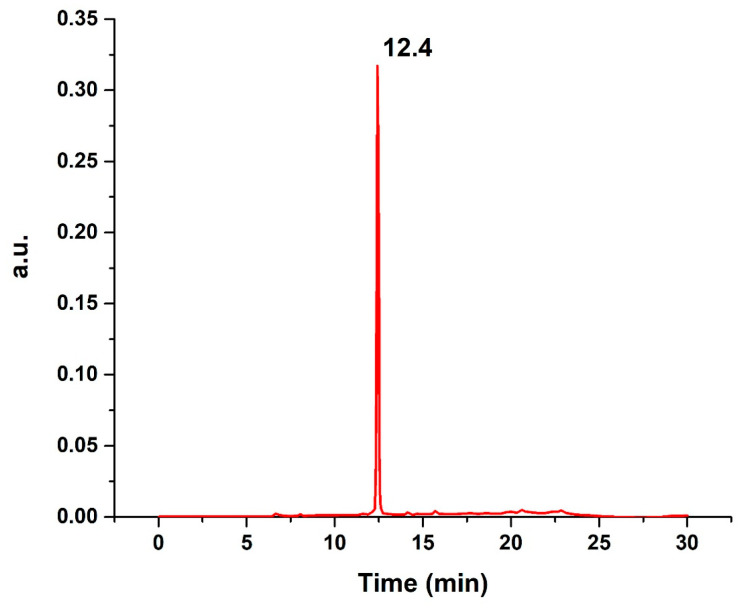
A reversed-phase HPLC system (Waters, Milford, CT, USA) with a PDA detector used a C-18 column (5 µm, 4.6 × 150 mm). The mobile phase consisted of 0.1% trifluoroacetic acid in water (pump A) and acetonitrile (pump B), with a flow rate of 1 mL/min. Elution involved: 100% A for 3 min, shifting to 50% A over 10 min and held for 10 min, then to 30% A over 3 min, and back to 100% A in 4 min.

The schematic structure of the novel DOTA–HYNIC–iPSMA–iGRP78 dimeric peptide ligand, with DOTA–HYNIC (2–hydrazinonicotinyl) at the center of the iPSMA and iGRP78 molecules connected via a 4-maleimidobutyric acid, which forms the branching point (4-(2,5-dioxopyrrolidin-1-yl)butanal), as well as its chemical characterization, are shown in Figure A1, Figure A2, Figure A3, Figure A4 and Figure A5. DOTA–HYNIC linked in the dimeric peptide was selected to enable future preparation of [^99m^Tc]Tc–HYNIC–iPSMA–iGRP78 as a theranostic pair for [^177^Lu]Lu–DOTA–HYNIC–iPSMA–iGRP78. The molecular docking of the dimeric peptide ligand was evaluated at the active sites of GRP78 (ATPase domain) and PSMA (binuclear zinc site), as described below.

### 2.1. Computational Simulation

**Figure 1:**
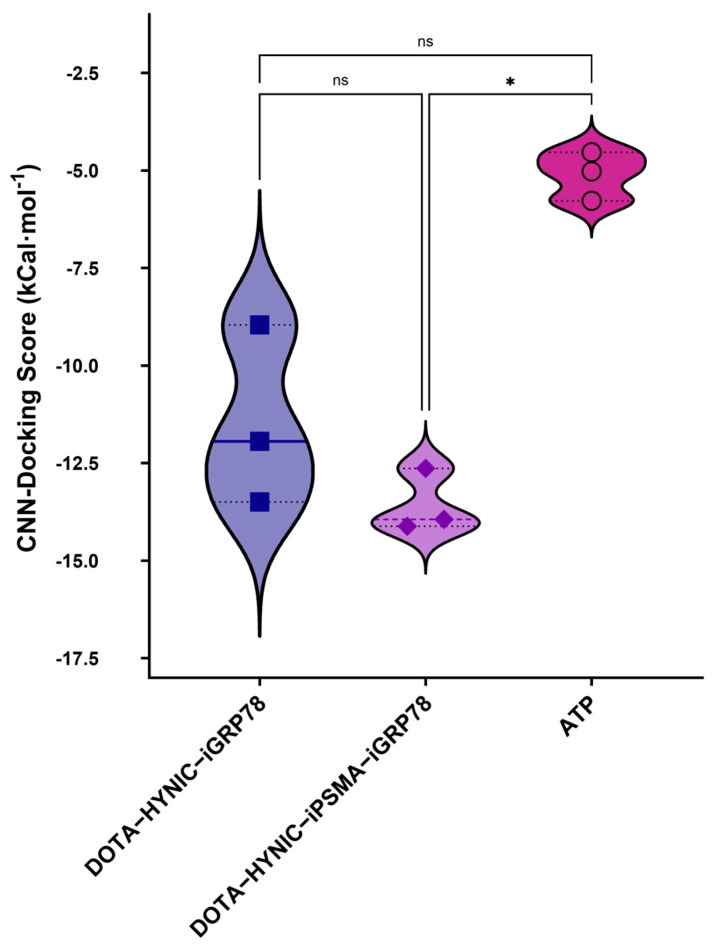
CNN-docking score of three inhibitor ligands in the active site of iGRP78 using the non-parametric Kruskal–Wallis test (* *p* < 0.05 statistically significant; ns: not statistically significant).

**Figure 2:**
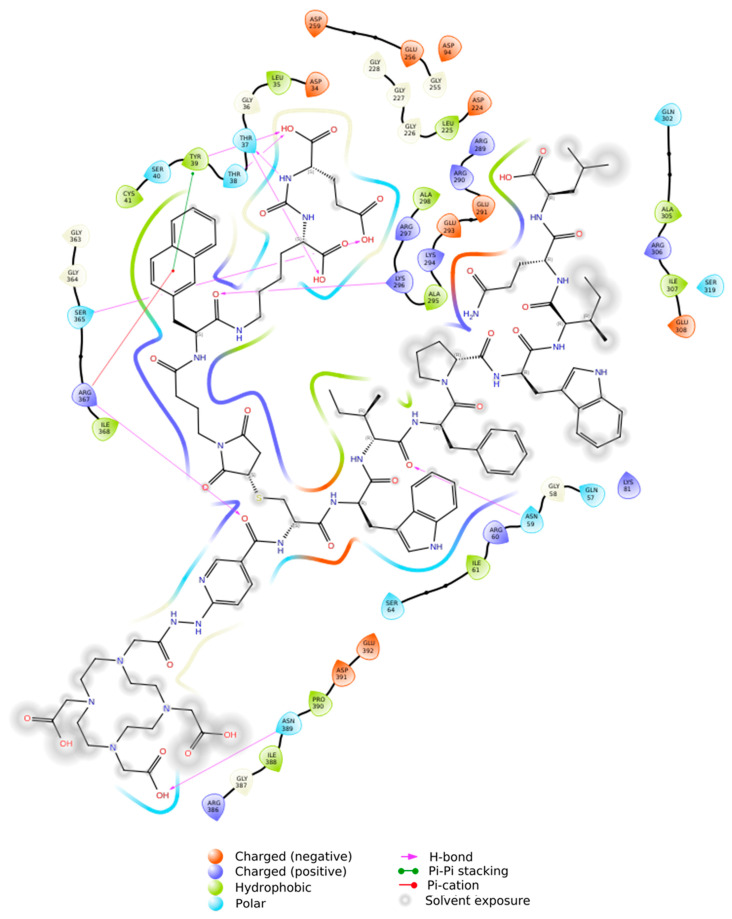
Two-dimensional diagram of the DOTA–HYNIC–iPSMA–iGRP78/GRP78 complex interactions.

The ensemble docking approach provided insights into the affinities and binding modes of the evaluated ligands compared to endogenous and synthetic controls. In the GRP78 system (PDB ID 6ASY), the reference ligand ATP showed high affinity for the GRP78 ATPase domain’s binding pocket (the catalytic site and target domain for inhibitor action), thereby confirming the model’s validity. Both DOTA–HYNIC–iGRP78 and DOTA–HYNIC–iPSMA–iGRP78 demonstrated more favorable convolutional neural network (CNN)-docking scores (−11 kcal·mol^−1^ and −14 kcal·mol^−1^, respectively) than ATP (−5.1 kcal·mol^−1^) (Table 1 and Figure 1), with the formation of a hydrogen bond network involving Lys296, Ser365, Arg367, as well as π–cation interactions with Arg367 (Figure 2). Arg367 has previously been identified by Bhattacharjee et al. [24] as a key GRP78 residue for inhibitor ligands. The authors provided the first structural analysis of GRP78 inhibition through small-molecule ATP-competitive ligands (EGCG and OSU-03012) using docking and 50 ns MDS where they focused exclusively on the isolated ATPase domain (PDB ID 3LDL) [24] and described key residues (Ile61, Glu293, Arg297, Arg367) as selectivity determinants relative to other HSP70 isoforms. In contrast, our study incorporates the complete GRP78 folding in both ATP-bound (PDB ID 6ASY) [25] and inhibitor-bound (PDB ID 5O5T) [26] conformations. This whole treatment allows simultaneous evaluation of nucleotide- and substrate-binding domains, capturing long-range interactions where possible allosteric effects can be captured during simulation times; either way, these interactions may also be absent in domain-restricted models. Bhattacharjee et al. [24] employed AutoDock Vina with empirical scoring reporting mean docking energies of −8.4 kcal·mol^−1^ (EGCG–GRP78) and −6.0 kcal·mol^−1^ (OSU–03012–GRP78). In the CNN-assisted ensemble docking, markedly stronger affinities were obtained for the conjugated ligands DOTA–HYNIC–iGRP78 (−11 kcal·mol^−1^) and DOTA–HYNIC–iGRP78–iPSMA (−14 kcal·mol^−1^).

A key finding to highlight in the coupling of DOTA–HYNIC–iPSMA–iGRP78 to the GRP78 protein is the lack of significant interactions with the iPSMA motif. In other words, the affinity of the heterodimeric peptide in GRP78 is due solely to the iGRP78 motif. This finding was corroborated by statistical analysis, which revealed no significant difference in the affinity score between the DOTA–HYNIC–iGRP78 monomer and the DOTA–HYNIC–iGRP78–iPSMA dimer (Figure 1 and Figure 2).

**Figure 3:**
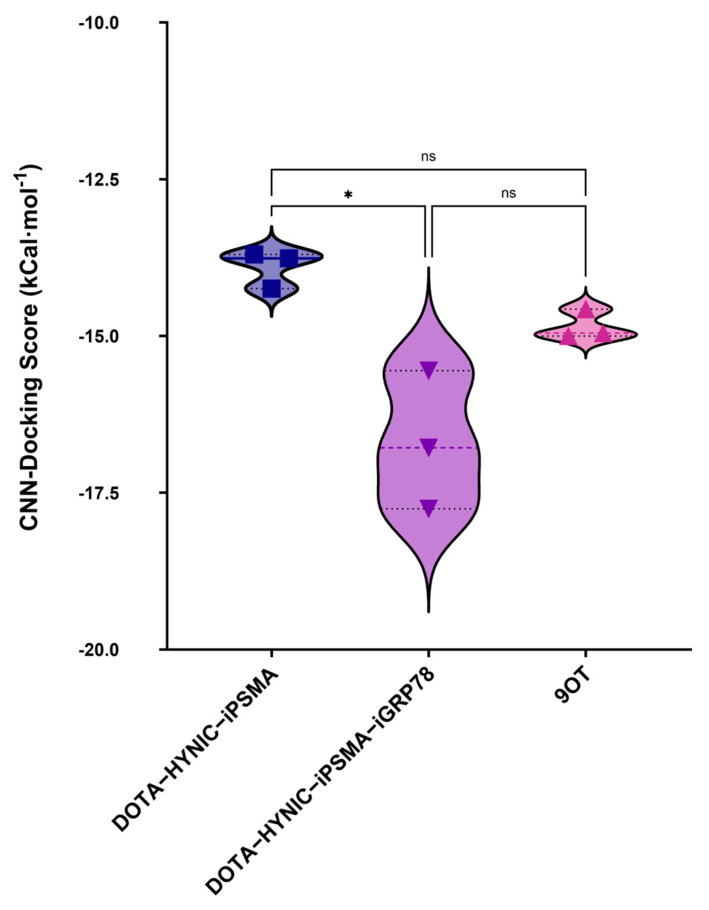
Comparative CNN-docking score of three inhibitor ligands in the active site of PSMA using the non-parametric Kruskal–Wallis test (* *p* < 0.05 statistically significant; ns: not statistically significant).

The binding mode of peptide ligands was also calculated for the PSMA protein (PDB ID 5O5T). The conjugates DOTA–HYNIC–iPSMA and DOTA–HYNIC–iPSMA–iGRP78 displayed good interaction and affinity profiles, with average binding energies of −13.9 kcal/mol and −17 kcal·mol^−1^, respectively. The latter was slightly higher than the PSMA1007 (9OT) reference ligand, with FDA approval for clinical use in the detection of primary and metastatic tumors expressing PSMA by molecular imaging (−15 kcal·mol^−1^) (Table 2). However, the Kruskal–Wallis test only confirmed significant differences (*p* < 0.05) in binding free energies between the modified dual-recognition dimeric molecule (DOTA–HYNIC–iPSMA–iGRP78) and the control monomer ligand (DOTA–HYNIC–iPSMA), but not between DOTA–HYNIC–iPSMA–iGRP78 and 9OT (Figure 3). Nevertheless, the results suggest improved binding stability and potential synergy between the iPSMA and iGRP78 motifs of the dimeric ligand in PSMA recognition compared to DOTA–HYNIC–iPSMA.

**Figure 4:**
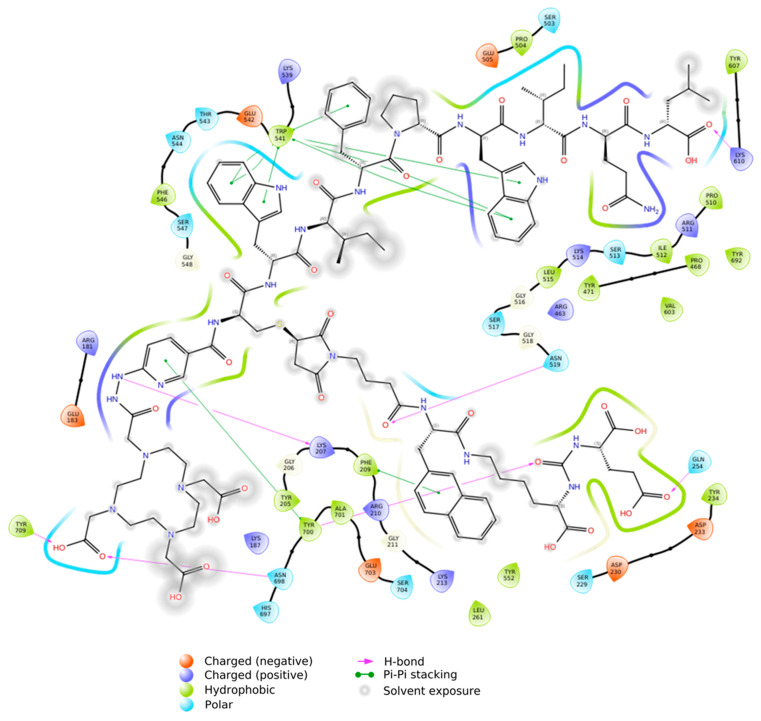
Two-dimensional diagram of the DOTA–HYNIC–iPSMA–iGRP78/PSMA complex interactions.

The interaction diagram of the DOTA–HYNIC–iPSMA–iGRP78/PSMA complex (Figure 4) revealed networks aligned with the protein’s functional domain, attributed to the interaction of urea in the –NH–CO–NH–Glu iPSMA site with Tyr700 and Gln254, as well as the WIFPWIQL (iGRP78) hydrophobic contacts with Trp541 (π-π) across the β-sandwich domain, which serve as a significant and additional anchoring site to PSMA. To validate the results related the synergic interaction of iGRP78 and iPSMA motifs in the PSMA protein, and enhance the static parameters obtained from docking, molecular dynamics simulation (MDS) was performed to examine the molecular flexibility and conformational changes of the Lu–DOTA–HYNIC–iPSMA–iGRP78 metallic coordination ligand under physiological conditions (solvent, ions, and temperature) over a period of 50 ns.

**Figure 5:**
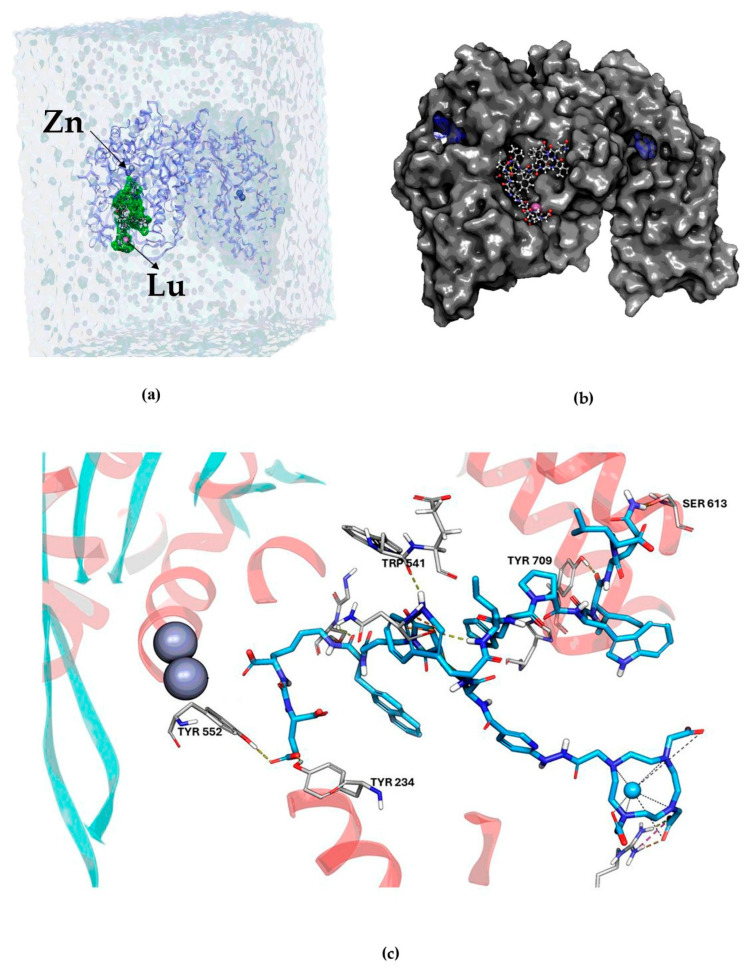
Molecular dynamics system: (**a**) simulation of the fully solvated Lu–DOTA–HYNIC–iPSMA–iGRP78/PSMA complex; (**b**) surface view of the PSMA protein with Lu–DOTA–HYNIC–iPSMA–iGRP78 in the binding site; and (**c**) 3D view of the Lu–DOTA–HYNIC–iPSMA–iGRP78 ligand interaction within the PSMA binding site. The protein is shown as a ribbon; the ligand (blue), involved residues (gray), Lu (light blue), and Zn (dark gray) are depicted as ball-and-stick.

The MDS of the fully solvated Lu–DOTA–HYNIC–iPSMA–iGRP78 complex, the surface view of the PSMA protein with Lu–DOTA–HYNIC–iPSMA–iGRP78 in the binding site, and the 3D ligand interactions within the PSMA binding site are shown in Figure 5. Notably, Figure 5c displays the iPSMA inhibitor sequence (–CH(COOH)–NH–CO–NH–Glu) interacting with Tyr552 and Tyr234, while being oriented toward the binuclear zinc in PSMA’s active site; additionally, Trp541 interacts with the iGRP78 sequence through the tryptophan indole side chain.

**Figure 6:**
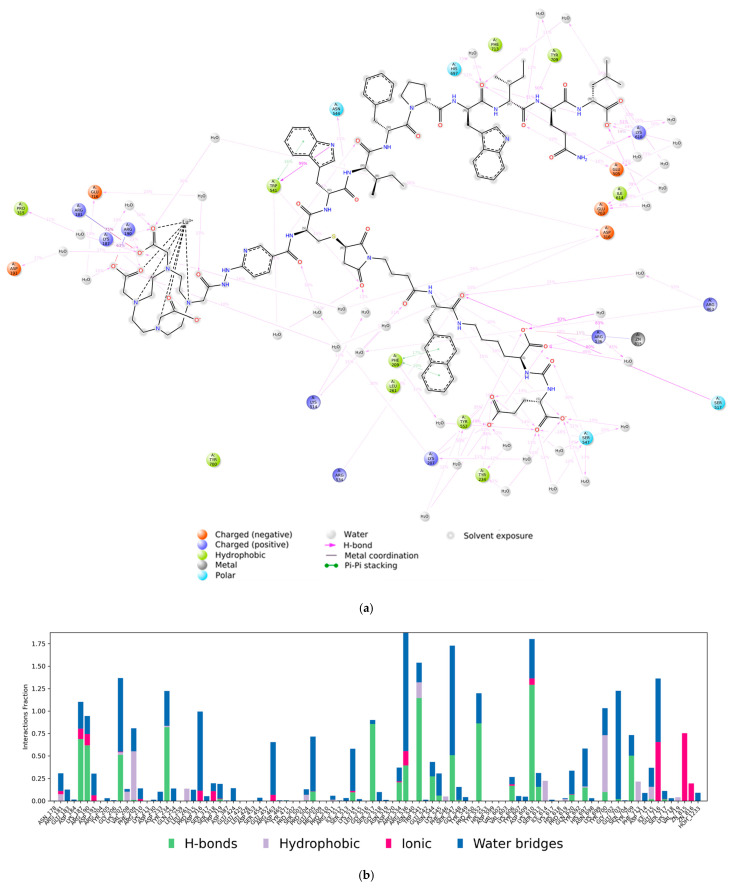
Molecular dynamics system: (**a**) 2D diagram of the Lu-DOTA–HYNIC–iPSMA–iGRP78/PSMA complex interactions (3 Å distance cutoff); (**b**) fractional occupancy of protein–ligand contacts between PSMA and Lu-DOTA–HYNIC–iPSMA–iGRP78 along the 50 ns simulation.

The 2D diagram of the Lu-DOTA–HYNIC–iPSMA–iGRP78/PSMA interactions (3 Å distance cutoff) confirmed the formation of hydrogen bonds involving the iPSMA sequence –CH(COOH)–NH–CO–NH–Glu with Ser547, Tyr552, Tyr234, and Arg536 (one of the arginine-rich patches in PSMA), which is coordinated to Zn815 (Figure 6a) [27]. Hydrogen bonds with Tyr709, Asn544, Lys610, and His697, as well as a π-π interaction with Trp541, were formed with the iGRP78 motif.

The MDS bar chart (Figure 6b) shows the bond strength of each PSMA protein residue with the Lu–DOTA–HYNIC–iPSMA–iGRP78 ligand and the type of bond formed between them. H–bonds, which are the most significant in the simulation due to their strength, have a fraction value above 1 for Trp541 and Lys610 because of their multiple contacts with the iGRP78 sequence. Trp541 also exhibited a hydrophobic interaction (π-π) fraction of 0.20 (20%) with the Trp–iGRP78 motif. The ionic interaction of Zn815, the active enzymatic center site of PSMA, with a fraction value of 0.75 (75%), was linked to the –CH(COOH)–NH–CO–NH–Glu iPSMA motif of the dual-recognition dimeric ligand.

**Figure 7:**
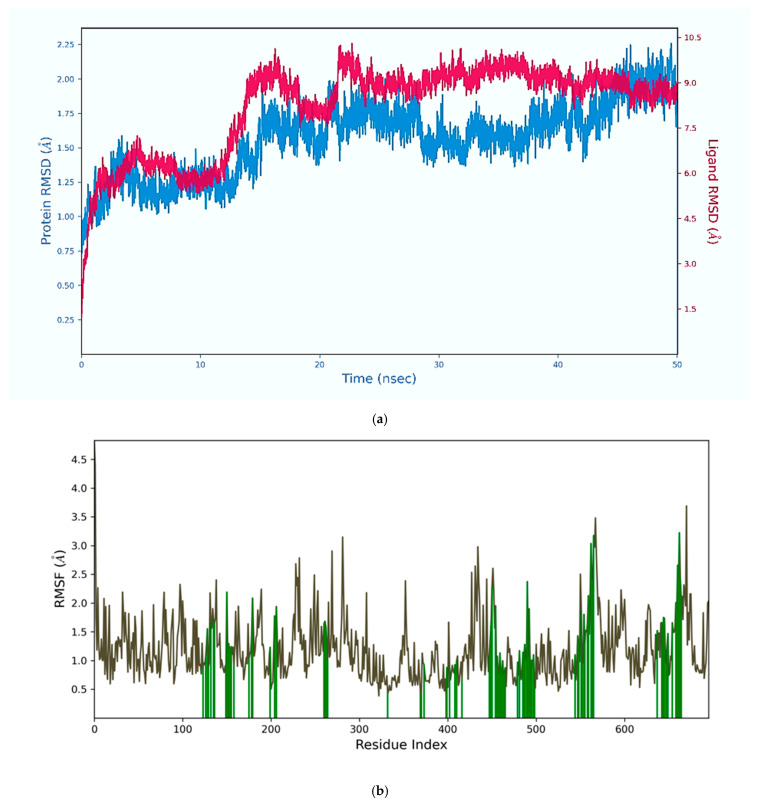
(**a**) Cα RMS (root mean square) Deviation of PSMA and Lu–DOTA–HYNIC–iPSMA–iGRP78 during the Molecular Dynamics simulation; (**b**) Cα root mean square fluctuation (RMSF) of PSMA residues (green) and their side chains (gray).

Root-mean-square deviation (RMSD), a measure of the average distance between alpha-carbon (Cα) atoms representative of the protein’s main chain backbone, remained below 2.25 Å between the crystal reference structure and the simulated global structure of the PSMA protein over time (Figure 7a). Furthermore, the RMS fluctuation (RMSF) of the Cα atoms also remained low (below 3 Å), indicating stability of the protein residues relative to their average positions throughout the MDS (Figure 7b). This suggests a good structural similarity and validates the reliability of the predicted interactions [28]. Similarly, the RMSD of the Cα atoms of the Lu–DOTA–HYNIC–iPSMA–iGRP78 ligand increased from 2 to 10 Å, and then it was reduced below 9 Å, indicating structural differences but also demonstrating that conformational fluctuations do not significantly affect coupling of the protein-ligand complex in the binding pocket (15 Å) over time (Figure 7a). It is important to note that, although an acceptable RMSD is less than 3 Å, this value depends on the ligand’s size and flexibility [29]. It is to be expected that a larger, more flexible ligand would have a higher RMSD compared to a smaller, rigid one. Furthermore, the RMSD graph is useful for demonstrating a stable trajectory, which indicates that the system has reached equilibrium. This was observed in Figure 7a with Lu–DOTA–HYNIC–iPSMA–iGRP78, which showed minimal fluctuations near the end of the simulation. Nevertheless, further studies should be performed considering different and longer molecular dynamics simulation times.

### 2.2. In Vitro Cellular Evaluation

[^177^Lu]Lu–iPSMA–iGRP78 and the controls [^177^Lu]Lu–iPSMA and [^177^Lu]Lu–iGRP78 used for in vitro cellular tests were obtained with a specific activity of 0.06 GBq/nmol and radiochemical purities exceeding 97%, which remain unchanged after 3 h of incubation at 37 °C in PBS or human serum, as assessed by radio-HPLC.

**Figure 8:**
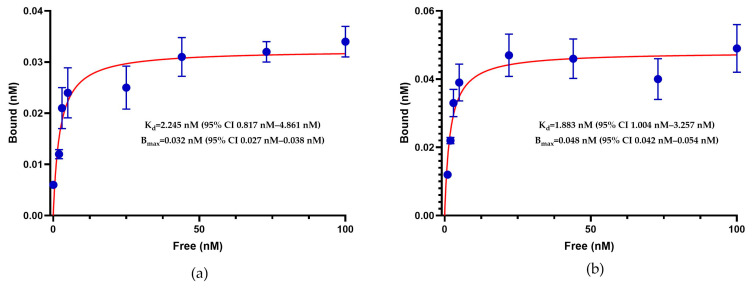
Saturation binding assay on LNCaP cells for (**a**) the monomer [^177^Lu]^nat^Lu–iPSMA and (**b**) the dimer [^177^Lu]^nat^Lu–iPSMA–iGRP78. The dissociation constant (K_d_) and maximum number of binding sites (B_max_) were determined using nonlinear regression analysis (GraphPad Prism).

The results of the saturation binding assays in LNCaP cells showed B_max_ values of 0.032 nM for [^177^Lu]Lu–iPSMA and 0.048 nM for [^177^Lu]Lu–iPSMA–iGRP78. In a biological context, the B_max_ values obtained for the monomeric and dimeric radiopharmaceuticals correspond to low receptor density or expression. Combined with low K_d_ values (e.g., <3 nM), this indicates high ligand specificity, since a small amount of the radioligand will attach even in the presence of fewer receptors (Figure 8).

**Figure 9:**
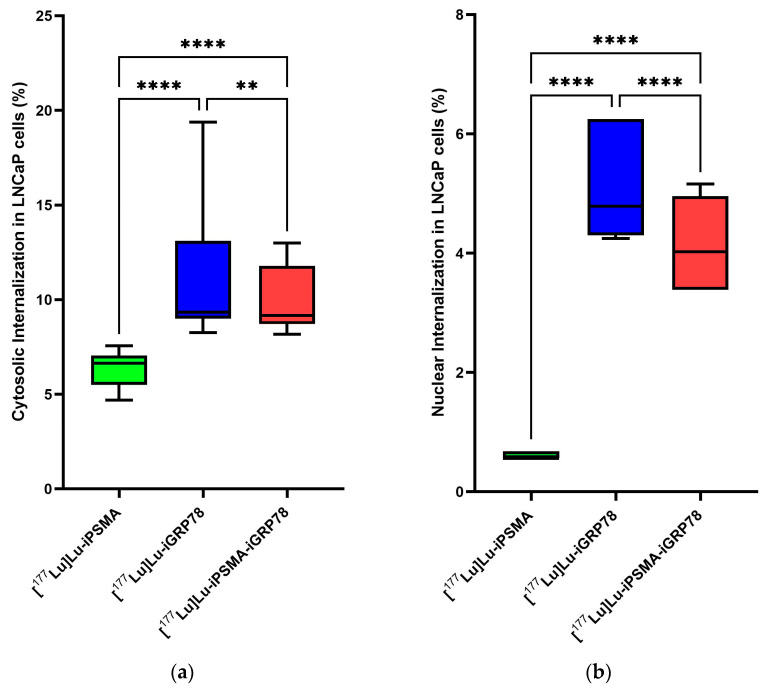
Percentage of (**a**) cytosolic and (**b**) nuclear internalization of [^177^Lu]Lu–iPSMA, [^177^Lu]Lu–iGRP78, and [^177^Lu]Lu–iPSMA–iGRP78 in LNCaP cells, which express PSMA and CS-GRP78 proteins. Two-way ANOVA with Tukey’s multiple comparisons test (α = 0.05) (** *p* = 0.0092 and **** *p* < 0.0001 statistically significant).

The saturation results also indicated a lower affinity for the monomer [^177^Lu]Lu–iPSMA (K_d_ = 2.245 nM) compared to the dimer [^177^Lu]Lu–iPSMA–iGRP78 (K_d_ = 1.883 nM). However, based on the confidence interval overlap criterion, the difference between the two K_d_ values was not statistically significant (Figure 8). In contrast, the subcellular biodistribution showed significant differences between monomeric and dimeric molecules in LNCaP cells that express PSMA and GRP78 (Figure 9). These differences were observed in both the percentage of cytosolic and nuclear internalization. The dimeric radiopharmaceutical achieved 10.44 ± 2.43% cytosolic internalization and 4.81 ± 0.94% nuclear internalization, while the [^177^Lu]Lu–iPSMA monomer showed 6.45 ± 0.60% cytosolic internalization and no uptake in the cell nucleus; and, surprisingly, with the highest internalization values associated with [^177^Lu]Lu–iGRP78 (Figure 9).

The localization of radiopharmaceuticals depends on the expression level of their targets, receptor turnover kinetics, and intracellular trafficking. Several studies have shown that PSMA internalization occurs rapidly (5 min) via clathrin-mediated endocytosis, involving both the endocytic and lysosomal pathways [5,30]. Internalization of PSMA in the absence of a ligand is slow, taking up to 2 h to reach 60%. Binding with ligands accelerates this process, but the rate of PSMA recycling depends on the type of ligand. Some, such as PSMA inhibitor peptides, hinder uncoupling and delay recirculation to the cell membrane [31]. PSMA has a cytoplasmic internalization sequence at the N-terminal end with the MXXXL motif (Methionine-X-X-X-Leucine), allowing it to internalize and persist in the cytosol when bound to [^177^Lu]Lu-iPSMA (Figure 9a), although a significant portion remains in the membrane. As PSMA has no nuclear localization signal, the radiopharmaceutical remains in the perinuclear area, which explains the non-cell nucleus accumulation of [^177^Lu]Lu-iPSMA (Figure 9b) [32].

The relocation of GRP78 in the cytosol, mitochondria, nucleus, and cell surface is linked to the state of the cells and affects cell signaling [33,34]. Its expression increases significantly in aggressive malignant cells, such as LNCaP and PC3, and correlates with the GRP78 expression observed in the immunofluorescence assay (Figure A8). Radioligands [^177^Lu]Lu-iGRP78 and [^177^Lu]Lu-iPSMA-iGRP78 showed a significantly higher uptake in cell cytoplasm and nucleus compared to [^177^Lu]Lu–iPSMA (Figure 9), with a subcellular distribution similar to GRP78 in the cytosol and nucleus of tumor cells [34,35,36]. In the dimer peptide internalization, a slight decrease was observed in both fractions regarding [^177^Lu]Lu-iGRP78, probably associated with the binding competition by the dual PSMA and GRP78 recognition.

**Figure 10:**
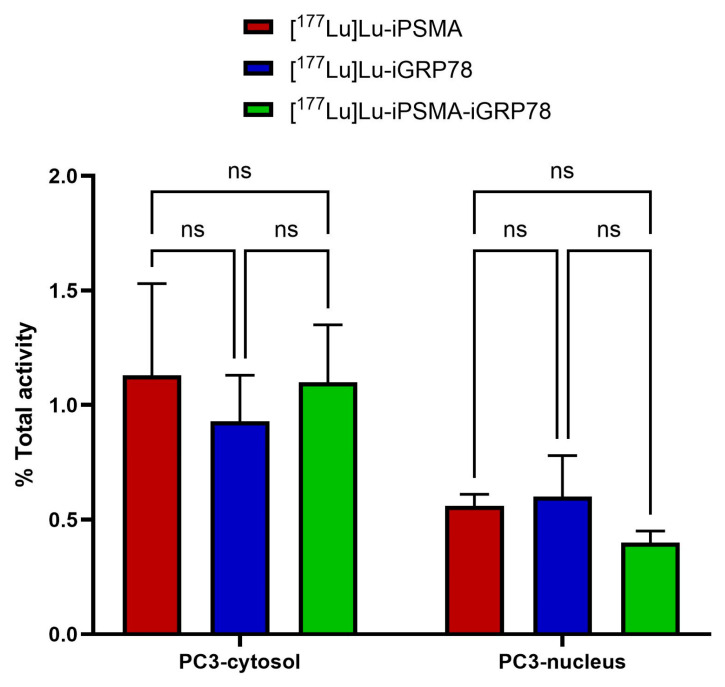
Percentage of cytosolic and nuclear internalization of [^177^Lu]Lu–iPSMA, [^177^Lu]Lu–iGRP78, and [^177^Lu]Lu–iPSMA–iGRP78 in PC3 cells, which do not express PSMA and CS-GRP78 proteins. Two-way ANOVA with Tukey’s multiple comparisons test (α = 0.05).

[^177^Lu]Lu–iPSMA, [^177^Lu]Lu–iGRP78, and [^177^Lu]Lu–iPSMA–iGRP78 showed low uptake in PC3 cells, both in the cellular cytosol and nucleus (Figure 10). Clathrin-mediated endocytosis, which is responsible for GRP78 trafficking, depends on the lipid enrichment of the cell membrane, which is a characteristic of prostate cancer cells [37]. Therefore, the uptake of GRP78-targeted radiopharmaceuticals was affected by variations in membrane composition between LNCaP and PC3 cells. For example, caveolin, which is present in PC3 cells but absent in LNCaP cells, forms a complex with GRP78 that regulates membrane trafficking, cell signaling, and responses to endoplasmic reticulum stress [38]. That is one reason that may explain why PC3 cells express high levels of cytosolic GRP78 but low levels of CS-GRP78, which, consequently, resulted in a low [^177^Lu]Lu-iGRP78 radiopharmaceutical uptake (Figure 10). Another possible explanation for the negligible uptake of [^177^Lu]Lu–iGRP78 and [^177^Lu]Lu–iPSMA–iGRP78 in PC3 cells, is that PC3 and LNCaP cells differ in their sensitivity to androgens and in their expression of androgen receptors. The PC3 cell line, classified as a model of androgen-independent metastatic prostate cancer with an aggressive and invasive phenotype, showed higher fluorescence intensity detection of GRP78 compared to LNCaP cells, which are considered indolent and androgen-dependent (Figure A8). Castration-resistant prostate cancer expresses a variant of the androgen receptor (ARV7), which binds to GRP78 and induces its degradation via the proteasome. This process can overcome androgen resistance [39].

**Figure 11:**
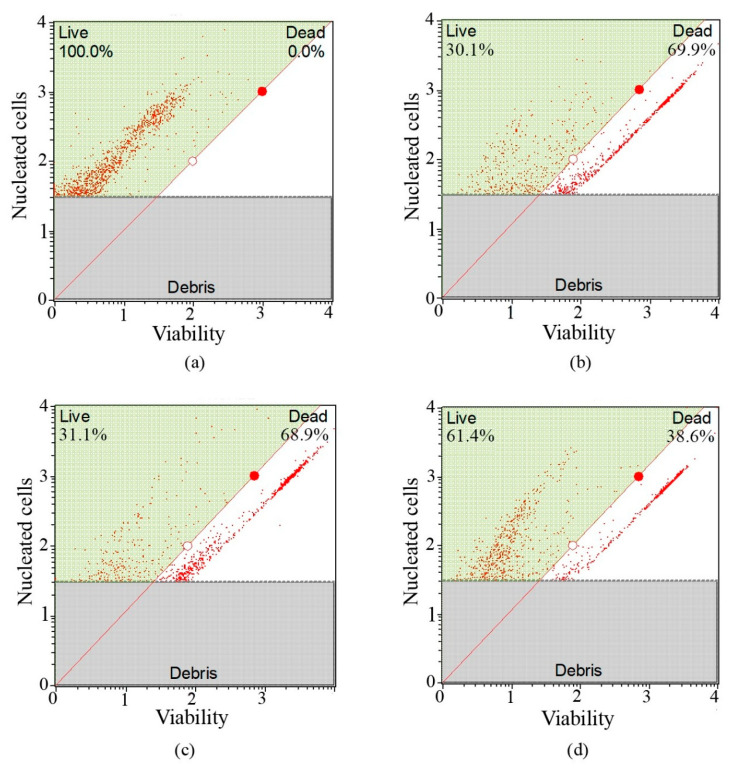
Viability profile histograms obtained by flow cytometry analysis of LNCaP cells that were treated with the following: (**a**) the control group (no treatment), (**b**) [^177^Lu]Lu-iPSMA-iGRP78, (**c**) [^177^Lu]Lu-iGRP78, and (**d**) [^177^Lu]Lu-iPSMA, under an acute radiation absorbed dose of 15 Gy/h for 5 h.

**Figure 12:**
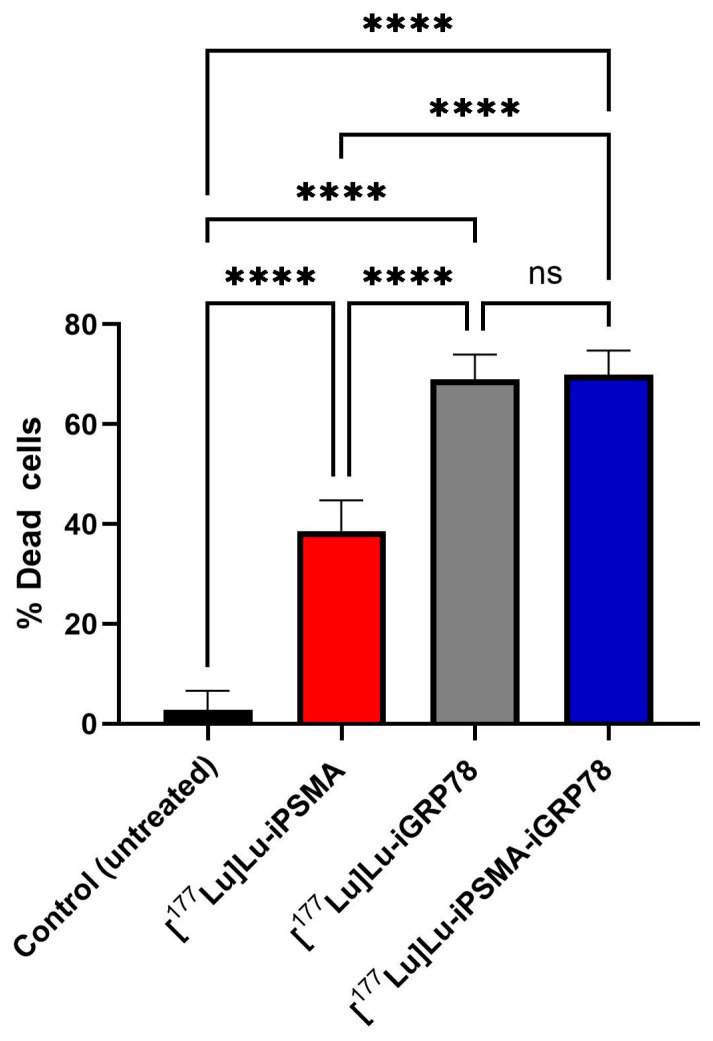
Cytotoxicity results of [^177^Lu]Lu-iPSMA, [^177^Lu]Lu-iGRP78, and [^177^Lu]Lu-iPSMA-iGRP78 on LNCaP cells obtained from the viability profiles (flow cytometry analysis) performed in triplicate (n = 3) from two independent experiments. Two-way ANOVA with Tukey’s multiple comparisons test (α = 0.05) (**** *p* < 0.0001 statistically significant).

The decrease in cell viability observed after acute treatment with radiopharmaceuticals correlated with their subcellular distribution (Figure 11). [^177^Lu]Lu–iGRP78 and [^177^Lu]Lu–iPSMA–iGRP78 exhibited a higher degree of internalization in both the cytosol and the nucleus compared to the monomer [^177^Lu]Lu–iPSMA, producing a high radiation absorbed dose to DNA. This resulted in cumulative biological damage, leading to the death of 69.93 ± 4.85% of cells treated with the dimeric radiopharmaceutical, with significant difference regarding [^177^Lu]Lu–iPSMA (38.63 ± 6.13% of dead cells) and without significant difference compared to that of [^177^Lu]Lu–iGRP78 (68.93 ± 5.05% of dead cells) (Figure 12).

Furthermore, it is likely that these radiopharmaceuticals stimulated the activation of apoptotic pathways by a mechanism analogous to that described for pyrotinib, recently reported by Bao et al. [40], where the interaction between GRP78 and EGFR in the endoplasmic reticulum induces the activation of programmed cell death in cancer cells.

It should be noted that EGFR expression in LNCaP cells is regulated by androgen receptors, whose degradation can be induced by their interaction with GRP78. Given that GRP78 acts as a cellular stress sensor, the radiation dose in the cell nucleus, resulting from the internalization of the radiopharmaceuticals, probably significantly increases cellular stress levels, thus promoting the overexpression of GRP78 and its translocation to the membrane (CS-GRP78). This phenomenon facilitates greater incorporation of the radiopharmaceutical, increasing the absorbed radiation dose and promoting interaction with key molecules involved in tumor progression at the metabolomic (EGFR, AR, integrins) and transcriptomic (c-Myc, HIF-1α) levels. All this forms a virtuous circle in which, as treatment progresses, conditions are created that enhance endocytosis and the efficacy of the dimeric radiopharmaceutical.

This research demonstrated that adding a GRP78 inhibitor to the [^177^Lu]Lu-iPSMA molecule is a novel and promising strategy for enhancing the internalization of molecularly targeted radiopharmaceuticals with the ability to accumulate in the cell nucleus. These preliminary data justify conducting preclinical studies to allow for complete characterization of the radiopharmaceutical. These studies should evaluate the internalization kinetics of [^177^Lu]Lu-iPSMA-iGRP78 in prostate cancer cells under various biochemical conditions, including hypoxia, endoplasmic reticulum (ER) stress, nutrient deprivation, growth factor presence, and radiation exposure. These studies will help determine changes in internalization rates, subcellular distribution, receptor turnover rates, ligand affinities, complex formation with other receptors, post-translational modifications of molecular targets, and increased expression and activation of signaling pathways (mytogenic, ADN repairing capacity, apoptotic, immunoregulatory, motility, angiogenic, resistance, and evasion). Additionally, it would be useful to determine the impact of various radiation doses mediated by ^177^Lu on the regulation of internalization kinetics, as well as the availability, activation status, and expression of CS-GRP78, GRP78, and PSMA receptors. The results regarding the high internalization of the monomeric radiopharmaceutical [^177^Lu]Lu-iGRP78 warrant a comprehensive study to determine its potential as a radiopharmaceutical for targeted radiotherapy.

## 3. Materials and Methods

### 3.1. Computational Simulation Methods

#### 3.1.1. Protein Preparation and Molecular Dynamics

The three-dimensional structures of the GRP78 protein and PSMA protein were retrieved from the Protein Data Bank under the PDB IDs 6ASY [25] and 5O5T [26], corresponding to the ATP-bound and PSMA1007 (9OT)-bound conformations, respectively. Both structures were prepared using the Protein Preparation Workflow in Schrödinger Maestro 2024-4 v14.2.118 (Schrödinger LLC, New York, NY, USA). Non-structural water molecules were removed, missing hydrogens were added, and protonation states were assigned at pH 7.4 using the PROPKA 2.0, algorithm [41].

#### 3.1.2. Ensemble Docking Protocol

Each protein structure was subsequently subjected to molecular dynamics simulations (MDS) using Maestro v2024-4 v14.2.118, MMshare Version 6.4.135, Release 2024-4, and Desmond Multisim v4.0.0 interoperability tools package under the OPLS-AA force field [42,43], to obtain a representative conformational ensemble. Simulations were performed under isothermal–isobaric (NPT) conditions at 310.15 K and 1 atm, using an octahedral water box of explicit TIP3P water molecules, which extended 12 Å from the solute and Na^+^/Cl^−^ counterions to neutralize the system charge. Representative frames were extracted at regular intervals throughout the trajectories and used for the ensemble docking protocol described below.

Ensemble docking was performed using GNINA 1.3 [44], which blends traditional scoring functions with convolutional neural networks. The Vinardo scoring function was used alongside the CNN_scoring = redock_default2018 model. For the 6ASY system, ATP served as the reference ligand. The docking grid was centered on the nucleotide-binding site at coordinates (center_x = 55.57, center_y = 4.88, center_z = −3.89) with dimensions of 25 × 25 × 25 Å^3^. For the 5O5T system, PSMA1007 (9OT) was the reference ligand, and the docking grid was set at (center_x = −16.38, center_y = 4.88, center_z = −33.42) with the same box dimensions.

For the GRP78–6ASY system, the ligands DOTA–hydrazinonicotinyl–Lys(Nal)–Urea–CWIFPWIQL (DOTA–HYNIC–iPSMA–iGRP78) and DOTA–hydrazinonicotinyl–CWIFPWIQL (DOTA–HYNIC–iGRP78) were docked together within the ATP binding site (reference ligand) using the following parameters: exhaustiveness = 64, num_modes = 6, cpu = 24, replicas = 3, and Monte Carlo search = enabled. For the GRP78–5O5T system, the ligands DOTA–HYNIC–iGRP78–iPSMA and DOTA–hydrazinonicotinyl–Lys(Nal)-Urea (DOTA–HYNIC–iPSMA) were tested against PSMA1007 (9OT) with exhaustiveness = 64, num_modes = 6, cpu = 24, replicas = 10, and the same convolutional neural network (CNN) scoring model.

All calculations were performed on a Linux-based workstation. The highest-scoring poses from each docking run were selected for subsequent structural and statistical analyses.

#### 3.1.3. Molecular Dynamics Simulation (MDS)

The best docked complex of iGRP78 was DOTA–HYNIC–iPSMA–iGRP78 (lowest CNN-docking score: −17 kcal/mol), which was used as initial coordinates for MDS to identify stable ligand-receptor binding modes. The simulations were conducted using the Maestro v2024-4 v14.2.118, MMshare Version 6.4.135, Release 2024-4, and Desmond Multisim v4.0.0 interoperability tools package under the OPLS-AA force field [42,43]. The complex system was solvated in octahedral boxes of explicit TIP3P water molecules, extending 15 Å from the solute, and neutralized with Na^+^ or Cl^−^ ions. All MDSs were carried out under periodic boundary conditions (PBC). Energy minimization involved 50,000 steps each of steepest descent and conjugate gradient, with positional restraints (10 kcal·mol^−1^·Å^2^) on solute heavy atoms. The system gradually heated from 0 to 310 K over 100 ps under constant volume, followed by 5 ns equilibration at 310 K using the Langevin thermostat and Berendsen barostat (1 atm, 2 ps relaxation), with restraints reduced over time. Production ran for 50 ns under NPT conditions with a 2 fs time step and hydrogen bond constraints; long-range electrostatics used a 10 Å cutoff. Trajectory analysis utilized Desmond-Maestro tools, focusing on alpha-carbon RMSD to evaluate structural flexibility.

#### 3.1.4. Structural and Statistical Analysis

Protein ligand interaction analyses were visualized using the Ligand Interaction Diagram module in the Maestro suite, and three-dimensional representations were generated within the same environment. CNN-Docking scores (kcal·mol^−1^) were compiled for the three ligands in each system, and the mean ± standard deviations were calculated. Statistical analyses were performed with GraphPad Prism v10.6.1, applying the non-parametric Kruskal–Wallis test to compare relative binding affinities among complexes. A *p* < 0.05 threshold was considered statistically significant.

### 3.2. Preparation of [^177^Lu]Lu–iPSMA–iGRP78

#### 3.2.1. Design, Synthesis, and Chemical Characterization

**Figure A6:**
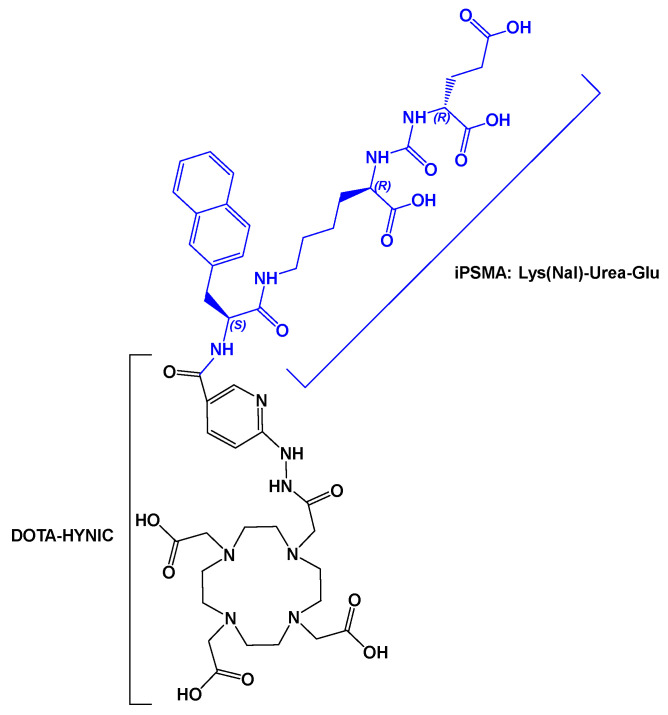
Schematic chemical structure of the DOTA-HYNIC–iPSMA monomeric peptide ligand.

**Figure A7:**
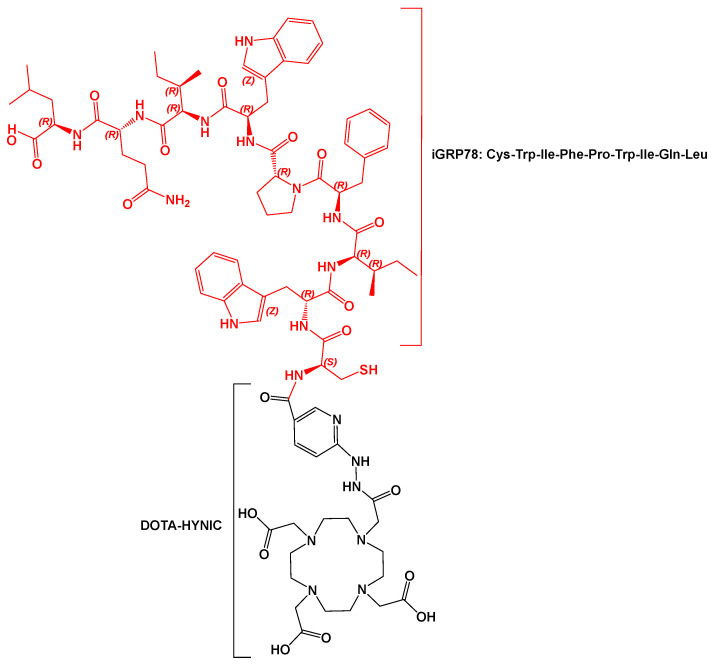
Schematic chemical structure of the DOTA-HYNIC–iGRP78 monomeric peptide ligand.

**Figure A8:**
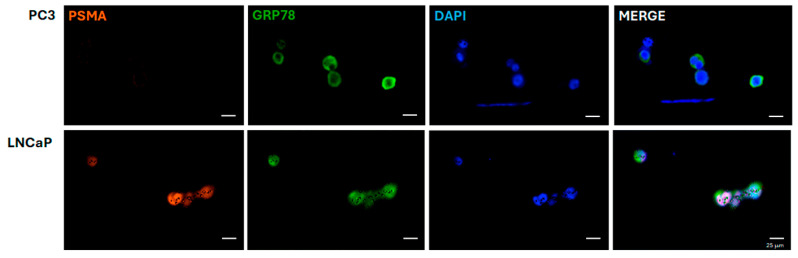
Microscopic fields (20×) of the expression in PC3 and LNCaP prostate cancer cells of PSMA (monoclonal anti-PSMA antibody, FOLH1/3734, Abcam) and GRP78 (polyclonal anti-GRP78 BIP antibody, ab21685, Abcam); the Hoechst-stained nucleus (DAPI), and the merged images.

The DOTA–HYNIC–iPSMA–iGRP78 peptide (Figure A1), with DOTA–HYNIC at the center of the iPSMA and iGRP78 monomers connected via a 4-maleimidobutyric acid, which forms the branching point (4-(2,5-dioxopyrrolidin-1-yl)butanal), was designed in the Laboratory on Research and Development of Radiopharmaceuticals at ININ, Mexico. The peptide was custom-synthesized by Yaxian Chemical Company (Shanghai, China). Chemical characterization using FT-IR, HPLC-MS, reversed-phase HPLC, and UV-Vis spectroscopy confirmed the peptide structure and purity (Figure A2, Figure A3, Figure A4 and Figure A5). For comparative purposes, DOTA–HYNIC–iPSMA (Figure A6) and DOTA–HYNIC–iGRP78 (Figure A7) were also prepared.

#### 3.2.2. Manufacturing of Multidose Lyophilized Kits

Lyophilized formulations were aseptically prepared by dissolving 12 µmol of DOTA–HYNIC–iPSMA–iGRP78, DOTA–HYNIC–iPSMA, or DOTA–HYNIC–iGRP78 in 20 mL DMSO/20% urea (2:1 *v*/*v*), stirring and heating at 90 °C. A solution of 2 g ascorbic acid and 1 g mannitol in 20 mL injectable water was added. After sterile filtration (0.22 µm), 2 mL aliquots were dispensed into 20 sterile ampoules for lyophilization. Separately, a 30 mL 1 M sodium acetate buffer (pH 5.0) was filtered, and 1.5 mL was dispensed into 20 sterile ampoules.

#### 3.2.3. Preparation of Radiopharmaceuticals

^177^LuCl_3_ (0.04 M HCl, 40 GBq/mL; ITM, Munich, Germany) was used to prepare [^177^Lu]Lu–iPSMA–iGRP78, [^177^Lu]Lu–iPSMA, and [^177^Lu]Lu–iGRP78 with multidose lyophilized kits under GMP conditions. To radiosynthesize, 1.5 mL of 1 M acetate buffer (pH 5.0) was added to ^177^LuCl_3_. Kits were reconstituted with sterile 0.5 mL DMSO followed by ^177^LuCl_3_ solution, then heated at 95 °C for 30 min. The final solution was diluted to 20 mL with injectable water, and dosing (7.4 GBq/4 mL) was performed using syringes via a GMP module (Musa GMP hot cell, Comecer, Ravenna, Italy).

#### 3.2.4. Quality Control

The quality control assessed bacterial endotoxins, sterility, pH, and appearance, following the general methods of the Mexican Pharmacopeia. Radiochemical purity was evaluated by reversed-phase radio-high-performance liquid chromatography (radioHPLC) ( LC-40DxR, Shimadzu, Kyoto, Japan). A shim-pack GIST C18 column (Shimadzu: 4.6 mm × 25 cm, 5 μm particle size) with a linear gradient from 100% to 10% of mobile phase A (0.1% TFA aqueous solution) and from 0% to 90% of phase B (0.1% TFA in acetonitrile) in 20 min at 1 mL/min. The retention time of [^177^Lu]Lu–iPSMA–iGRP78 was 17.4 ± 0.3 min, and 3.7 ± 0.2 min for ^177^LuCl_3_. Radiochemical purities of [^177^Lu]Lu–iPSMA (t_R_ = 15.2 ± 0.2 min) and [^177^Lu]Lu–iGRP78 (t_R_ = 19.2 ± 0.4 min) were also verified to be higher than 95%.

#### 3.2.5. Stability in PBS and Human Serum

To assess the in vitro stability of [^177^Lu]Lu-iPSMA-iGRP78, 10 µL (18.5 MBq) of the radiopharmaceutical was diluted in either 1 mL of PBS or 1 mL of human serum (NIST-909c certified human serum sample; Sigma-Aldrich, St. Louis, MO, USA) (n = 3). The mixtures were incubated for 3 h at 37 °C. Then, 0.5 mL of acetonitrile was added to the human serum samples to precipitate the proteins, and the mixtures were centrifuged at 500× *g* for 10 min. The radiochemical purity of the human serum supernatants and PBS solution samples was analyzed by reverse-phase radio-HPLC, as described above.

### 3.3. Cellular Internalization Measurement of [^177^Lu]Lu-iPSMA-iGRP78 in Prostate Cancer Cell Lines

#### 3.3.1. Cell Lines

PC-3 (CRL-1435; ATCC, Manassas, VA, USA) and LNCaP human prostate cancer cells (CRL-1740; ATCC, USA) were cultured in Roswell Park Memorial Institute medium (RPMI; Sigma-Aldrich, USA) at 37 °C, 85% humidity, 5% CO_2_, and 10% fetal bovine serum.

Assays were performed to evaluate the cellular uptake and subcellular distribution of [^177^Lu]Lu–iPSMA–iGRP78 and the control monomers [^177^Lu]Lu–iPSMA and [^177^Lu]Lu–iGRP78 in two prostate cancer cell lines: LNCaP (PSMA+, SC-GRP78+) [45] and PC3 (PSMA^−^, CS-GRP78^−^) [46]. Cells (1 × 10^5^/200 µL) were incubated with the radiopharmaceutical (37 kBq/4 nmol) for 1 h at 37 °C to assess the distribution between the cytoplasm and nucleus.

#### 3.3.2. Saturation Binding Assay

The affinity comparison between the [^177^Lu]Lu–iPSMA–iGRP78 dimer and [^177^Lu]Lu–iPSMA monomer was performed in LNCaP cells. For the saturation binding analysis, cells were seeded on 96-well plate cultures (1 × 10^6^ cells/well) and incubated at 37 °C under a CO_2_ atmosphere for 24 h, then the plates were placed on ice for 30 min. Next, they were incubated at 4 °C for 2 h with increasing concentrations (from 1nM to 100 nM) of the monomer [^177^Lu]^nat^Lu-iPSMA or the dimer [^177^Lu]^nat^Lu-iPSMA-iGRP78. Finally, cells were washed with PBS followed by the addition of 1M NaOH for cell lysis. This solution was measured for radioactivity using a NaI(Tl) gamma counter (NML Inc., Littleton, CO, USA). Non-specific binding of [^177^Lu]Lu–iPSMA–iGRP78 and [^177^Lu]Lu–iPSMA was determined under the same experimental conditions described above but in the presence of PMPA (2-(phosphonomethyl) pentanedioic acid) at a final concentration of 1 μM. The dissociation constant (K_d_) and maximum number of binding sites (B_max_) were calculated by a lineal regression analysis with data (n = 3) from two independent experiments using the GraphPad Prism software (v. 10.6.1).

#### 3.3.3. GRP78 and PSMA Cell Immunodetection

For the immunofluorescence assay, 3 × 10^4^ LNCaP or PC3 cells adhered to a well Nunc Lab-Tek Chamber Slide System, were incubated for 30 min in a 4% PFA fixing solution and permeabilized with a 0.5% detergent solution based on triton X-100 before avoiding unspecific antibody binding by incubation with a 5% BSA-V (A3059, Millipore Sigma, Burlington, MA, USA) blocking solution for 30 min at room temperature. For protein detection an antibody cocktail solution in 1% BSA containing polyclonal anti-GRP78 BIP antibody 1:120 (ab21685, Abcam, Cambridge, UK) and monoclonal anti-PSMA antibody (FOLH1/3734, Abcam) 1:100 was prepared, and blockade cells were incubated with it overnight at 4 °C. After five washes, the cells were incubated for 1 h with a mixed solution containing the Goat anti-Rabbit igG (h + L) Highly Cross-Adsorbed Secondary Antibody, Alexa Fluor Plus 488 1:1000 (A32731, Invitrogen, Waltham, MA, USA) and Donkey anti-Mouse IgG (H + L) Highly Cross Adsorbed Secondary Antibody, Alexa Fluor 594 1:1000 (A-21203, Invitrogen) for 1 h at room temperature in the dark. Finally, slides were mounted and the cell nuclei stained with Fluormount-G (Invitrogen) and observed under an inverted epi-fluorescent microscope (Meiji Techno Co., Ltd., Saitama, Japan).

#### 3.3.4. Separation of Cytoplasmic and Nuclear Fractions

Following incubation, tubes were measured with a NaI(Tl) crystal scintillation gamma-counter (NML Inc., TX, USA) to determine 100% of the initial activity. The tubes were centrifuged at 250× *g* for 5 min, and the supernatant was withdrawn. The activity in the pellet was measured to determine the percentage of cellular uptake (surface uptake) relative to the initial activity. Next, the nuclei and cytoplasm of the cells were separated using a Nuclear Extraction Kit (Chemicon International, Inc., Temecula, CA, USA). To prepare for fractionation, the cells were first washed with 1 mL of 0.2 M acetic acid/0.5 M NaCl. The tubes were centrifuged at 250× *g* for 5 min, washed with phosphate-buffered saline (PBS), and then centrifuged again to ensure thorough removal of extracellular components.

#### 3.3.5. Measurement of Internalized Activity

After centrifugation, the cell pellet, which corresponds to the internalized activity, was quantified using a gamma counter. The radioactivity measured in the pellet was defined as 100% of the internalized activity.

The pellet was resuspended in cytoplasmic lysis buffer with dithiothreitol and a protease inhibitor cocktail to maintain protein stability during lysis. Mechanical disruption was performed by passing the suspension through a 27-gauge needle multiple times using a syringe to ensure complete cell breakdown. The resulting lysate underwent centrifugation at 8000× *g* for 20 min at 4 °C. The supernatant, representing the cytosolic fraction, was collected and analyzed for radioactivity via gamma counting.

The remaining pellet, containing the nuclear fraction, was resuspended in nuclear extraction buffer supplemented with dithiothreitol and a protease inhibitor cocktail. Nuclear disruption was carried out before centrifuging at 16,000× *g* for 5 min at 4 °C. The supernatant contained the nuclear extract, while the membrane components remained in the pellet.

Radioactivity measurements were conducted on both the nuclear extract and the membrane pellet fractions using a gamma counter. The percentage of activity within each subcellular compartment was calculated for each time point, allowing for evaluation of the distribution of [^177^Lu]Lu–iPSMA–iGRP78 and control monomers [^177^Lu]Lu–iPSMA and [^177^Lu]Lu–iGRP78 between the cytoplasmic and nuclear compartments of prostate cancer cells over time.

### 3.4. Cytotoxicity Assessment of [^177^Lu]Lu-iPSMA-iGRP78 in Prostate Cancer Cell Lines

The effects of radioligands on LNCaP cell viability were evaluated using advanced flow cytometry techniques. The radioligands tested were [^177^Lu]Lu–iPSMA–iGRP78, [^177^Lu]Lu–iPSMA, and [^177^Lu]Lu–iGRP78. This evaluation was conducted to assess their impact on the survival of prostate cancer cells. Cell viability was measured using the Count & Viability kit (Muse^®^). Prior to analysis, the Muse^®^ cell analyzer (Merck Millipore, Burlington, MA, USA) was calibrated with the verification kit to ensure its accuracy. First, a fresh, untreated LNCaP cell sample was analyzed to establish reference parameters, including cell size, granularity, and natural cell viability. To accurately distinguish between live and dead cells, an aliquot of the same cell population was treated with 0.05% Triton X-100. This treatment defined the dead cell detection window in the analyzer. Next, a specific detection window was established for LNCaP cells to differentiate between viable and non-viable cells during analysis accurately. For each experimental group, a cell suspension containing 5 × 10^4^ cells in 200 µL was prepared. The cells were then treated as follows: 1.4 MBq of (a) [^177^Lu]Lu–iPSMA–iGRP78, (b) [^177^Lu]Lu–iPSMA, (c) [^177^Lu]Lu–iGRP78, and (d) untreated. After five hours of treatment (15 Gy/h at whole-cell dose), the cells were mixed with 450 µL of viability reagent and incubated for 5 min at room temperature in the dark. All measurements were performed in triplicate (n = 3) from two independent experiments to ensure reliability and reproducibility. To determine the radiation absorbed dose rate to the whole cell (15 Gy/h), the total number of nuclear transformations (N) was calculated. This value was normalized to the administered activity unit (MBq·s/MBq) by integrating the activity (A(t)) from time zero to five hours. Next, the absorbed dose was calculated by multiplying N by the dose factor (DF: from cell to cell) (Gy/Bq·s), as determined by MIRDcell software, considering the crossfire absorbed dose (version 2.1).

## 4. Conclusions

The results of this research demonstrated that conjugating a GRP78 inhibitor to the radiotherapeutic [^177^Lu]Lu–iPSMA improves cytosolic internalization in prostate cancer cells, compared to the monomer [^177^Lu]Lu–iPSMA. Molecular docking and molecular dynamics results indicated that the dimeric ligand [^177^Lu]Lu–iPSMA–iGRP78 has a higher affinity for both GRP78 and PSMA, suggesting a synergistic effect on recognition and binding stability. In vitro assays confirmed that the dimeric radiopharmaceutical achieves greater internalization in the cytosol and nucleus of LNCaP (PSMA+, GRP78+) cells, resulting in greater cytotoxicity and therapeutic potential. Furthermore, the selectivity observed in cell lines that do not express both receptors (PC3) highlight the specificity of the compound, minimizing unwanted uptake. These findings suggest that the design of bispecific radiotherapeutics targeting PSMA and GRP78 may represent a promising strategy to improve the efficacy of targeted therapy in castration-resistant prostate cancer. The preliminary results obtained justify additional preclinical studies, both in vitro and in vivo, to validate the potential usefulness of [^177^Lu]Lu–iPSMA–iGRP78 in improving targeted radiotherapy.

## Figures and Tables

**Table 1:** Convolutional neural network (CNN)-docking score and the negative logarithm of the inhibition constant (pKi) (affinity score) of three inhibitor ligands in the active site of GRP78.

Ligands	CNN-Docking Score Mean (kcal·mol^−1^) [SD]	CNN Affinity Mean (pKi) [SD]
DOTA-HYNIC-iGRP78	−11.0 [2.3]	9.4 [0.5]
DOTA-HYNIC-iPSMA-iGRP78	−14.0 [0.8]	10 [0.4]
ATP	−5.1 [0.6]	8.5 [0.1]

**Table 2:** Convolutional neural network (CNN)-docking score and the negative logarithm of the inhibition constant (pKi) (affinity score) of three inhibitor ligands in the active site of PSMA.

Ligands	CNN-Docking Score Mean (kcal·mol^−1^) [SD]	CNN Affinity Mean (pKi) [SD]
DOTA-HYNIC-iPSMA	−13.9 [0.3]	10.2 [0.14]
DOTA-HYNIC-iPSMA-iGRP78	−17.0 [1.1]	11.5 [0.23]
9OT	−15.0 [0.2]	11.0 [0.24]

## Data Availability

Data is contained within this article.

## Abbreviations

The following abbreviations are used in this manuscript:

DAPI	4′,6-diamidino-2-phenylindole
DOTA	1,4,7,10-tetraazacyclododecane-N,N′,N″,N‴-tetraacetic acid
FT-IR	Fourier-transform infrared spectroscopy
GRP78	Glucose-regulated protein 78
HYNIC	2–hydrazinonicotinyl
HPLC	High-performance liquid chromatography
iGRP78	Glucose-regulated protein 78 inhibitor
iPSMA	Prostate-specific membrane antigen inhibitor
LNCaP	Prostate cancer cell line (LNCaP)
PDA	Photodiode array detector
PC3	Prostate cancer cell line (PC3)
PSMA	Prostate-specific membrane antigen
CS-GRP78	Surface cell glucose-regulated protein 78
UPLC	Ultra-performance liquid chromatography
UV-Vis	Ultraviolet–visible spectroscopy

## Appendix A

Schematic chemical structure of the novel DOTA-HYNIC–iPSMA–iGRP78 ligand (Figure A1) and its chemical characterization by FT-IR (Figure A2), UV-Vis (Figure A3), mass spectroscopy (Figure A4), and reversed-phase HPLC (Figure A5) are shown below.
